# Evoked Potentials Differentiate Developmental Coordination Disorder From Attention-Deficit/Hyperactivity Disorder in a Stop-Signal Task: A Pilot Study

**DOI:** 10.3389/fnhum.2021.629479

**Published:** 2021-03-11

**Authors:** Emily J. Meachon, Marcel Meyer, Kate Wilmut, Martina Zemp, Georg W. Alpers

**Affiliations:** ^1^Department of Psychology, School of Social Sciences, University of Mannheim, Mannheim, Germany; ^2^Department of Psychology, University of Konstanz, Konstanz, Germany; ^3^Department of Psychology, Health and Professional Development, Oxford Brookes University, Oxford, United Kingdom; ^4^Department of Clinical and Health Psychology, University of Vienna, Vienna, Austria

**Keywords:** dyspraxia, executive function, response inhibition, electroencephalography, ERPs

## Abstract

Developmental Coordination Disorder and Attention-Deficit/Hyperactivity Disorder are unique neurodevelopmental disorders with overlaps in executive functions and motor control. The conditions co-occur in up to 50% of cases, raising questions of the pathological mechanisms of DCD versus ADHD. Few studies have examined these overlaps in adults with DCD and/or ADHD. Therefore, to provide insights about executive functions and motor control between adults with DCD, ADHD, both conditions (DCD + ADHD), or typically developed controls, this study used a stop-signal task and parallel EEG measurement. We assessed executive performance via go accuracy and go reaction time, as well as motor response inhibition via stop-signal reaction time. This was complemented with analysis of event-related potentials (ERPs). Based on existing investigations of adults with DCD or ADHD, we expected (1) groups would not differ in behavioral performance on stop and go trials, but (2) differences in ERPs, particularly in components N200 (index of cognitive control) and P300 (index of attention and inhibition) would be evident. The sample included *N* = 50 adults with DCD (*n* = 12), ADHD (*n* = 9), DCD + ADHD (*n* = 7), and control participants (*n* = 22). We replicated that there were no between-group differences for behavioral-level executive performance and motor response inhibition. However, on a physiological level, ERP components N200 and P300 differed between groups, particularly during successful response inhibition. These ERPs reflect potential endophenotypic differences not evident in overt behavior of participants with ADHD and/or DCD. This suggests a disorder specific employment of inhibition or general executive functions in groups of adults with DCD, DCD + ADHD, ADHD, or control participants.

## Introduction

Developmental Coordination Disorder (DCD) and Attention-Deficit/Hyperactivity Disorder (ADHD) are lifelong neurodevelopmental disorders known to co-occur in up to 50% of cases (Blank et al., 2019). The primary symptoms of DCD are difficulties in learning and executing coordinated fine and gross movements, while primary symptoms of ADHD include inattention, hyperactivity, and impulsivity (American Psychiatric Association, 2013). In the last decade, evidence for substantial symptomatic overlaps between the two disorders has been observed. This includes children with DCD displaying hyperactivity (Harrowell et al., 2018), and having deficiencies in executive functions (Bernardi et al., 2015; Sartori et al., 2020) with the latter potentially persisting overtime (Bernardi et al., 2018; Wilson et al., 2020). These are typically observed as core symptoms of ADHD. Conversely, impaired fine and gross motor skills, a primary symptom of DCD, have been found for children with ADHD in comparison to typically developing individuals (Kaiser et al., 2015). This overlap is not limited to children, as, for instance, adults with DCD have also expressed difficulties with executive functions (e.g., Tal Saban et al., 2014), while those with ADHD have shown weakened visuo-motor adaptation (Kurdziel et al., 2015). Despite considerable overlap between DCD and ADHD, researchers have often supported the notion that they are unique disorders (e.g., Martin et al., 2006; Sergeant et al., 2006; Goulardins et al., 2015). In their critical review, Goulardins et al. (2015) pointed out that further research is needed to identify the possible sources of symptomatic overlap in DCD and ADHD. This research gap was also documented in a recent international consensus on DCD, in which the authors also highlight a generally growing body of research on adult populations (Blank et al., 2019). To expand on this literature, a better understanding of the mechanisms involved in executive functions and motor skills for adults with DCD versus ADHD is pertinent.

An obvious target for such research is to examine how inhibitory control and related underlying mechanisms differ in DCD and ADHD. First, inhibitory control is central to executive functioning (Miyake and Friedman, 2012; Matzke et al., 2018). Second, inhibitory deficits have often been observed in those with ADHD compared to typically developing individuals, with some evidence also emerging for DCD (Wodka et al., 2007; DCD versus controls: Bernardi et al., 2015; Sartori et al., 2020). Given the prominence of work on response inhibition with ADHD (e.g., Pauli-Pott and Becker, 2011) along with a dearth of work on inhibition more generally for DCD, this is an apt starting point for examining unique features of inhibition between both conditions. Thus, the purpose of this paper is a unique and necessary investigation of the differences in motor inhibition between adults with DCD and ADHD and both conditions.

To this end, we used the Stop-Signal Task (SST), as it places particularly high demands on motor response inhibition (Rubia et al., 2001). Arguably, such increased demands on inhibitory performance should render the SST sufficiently sensitive to reveal capacity limits in inhibitory control (i.e., avoid the risk of a ceiling effect with optimal inhibitory performance), and thus permit observing differential effects across groups. Nonetheless, potential differences between DCD and/or ADHD could go undetected at the behavioral level alone (Mandich et al., 2003; He et al., 2018a). Therefore, we included parallel neurophysiological measurement. Event-related P300 and N200 components were examined at the neurophysiological level based on their high relevance in inhibitory control in previous research of the SST, especially with ADHD versus control groups (e.g., Bekker et al., 2005; MacLaren et al., 2007; Senderecka et al., 2012). We examine differences in these components between groups of adults with DCD, ADHD, both conditions, and those of typical development.

### Behavioral Performance: Response Inhibition in DCD and ADHD

The SST is an opportune method for a closer look into inhibitory control and related executive processes (e.g., attention, Miyake and Friedman, 2012; Matzke et al., 2017, 2018), relevant to several disorders (e.g., ADHD; Verbruggen and Logan, 2008b; Nigg, 2017). The SST typically involves an ongoing binary selection process across go trials (e.g., left or right). On a small number of trials, a stop-signal cues participants that the response they are about to execute should be inhibited (stop trial). Stop trials involve a brief presentation of a go stimulus before the stop-signal appears after a variable delay. Owing to this delayed signal onset, the SST measures top-down response inhibition rather than automatic inhibition (Verbruggen and Logan, 2008a).

Various forms of the SST have been used to compare and contrast individuals with ADHD to unaffected individuals (e.g., Rubia et al., 2005; MacLaren et al., 2007; Pauli-Pott and Becker, 2011; Senderecka et al., 2012; Congdon et al., 2014). It has even been considered that impairments in task performance among those with ADHD versus typically developing individuals on a SST could be indicative of a prefrontal lobe dysfunction (Homack and Riccio, 2004), however, this may be specific to child populations. There is some evidence for potentially impaired inhibition in those with DCD compared to typically developing individuals as well (e.g., Mandich et al., 2003; He et al., 2018a) but when adults with DCD performed worse, these differences were subtle. One study has recently examined SST performance in a group of young adults with DCD versus typically developing individuals and found only a trend toward significantly different stop-signal reaction times (SSRTs; He et al., 2018a). This study also examined Go/No-Go task performance (automatic inhibition) (Verbruggen and Logan, 2008a), and found the DCD group had significantly reduced performance compared to typically developing individuals, showing some inhibitory differences in adults with DCD at the behavioral level (He et al., 2018a). More research is needed to determine if these inhibitory differences are consistent for adults with DCD and/or ADHD. Therefore, in the present study, we used the SST to capture the top-down processing of motor response inhibition for insight into executive functioning differences at both behavioral and neural levels.

### ERP Evidence for Inhibitory Differences in ADHD and DCD

Adults with DCD and ADHD may employ advanced learned compensatory mechanisms (Kysow et al., 2017; Wilmut, 2017) which may in turn obscure their true differences based on overt behavior alone. Therefore, it is important to consider the diverse endophenotypes of DCD and ADHD with parallel neurophysiological assessment. Endophenotypes, which are sometimes referred to as mechanisms, are the processes by which a phenotype is expressed (Rommelse et al., 2008). There is little research that has examined potential differences in both behavior and endophenotypic expressions (e.g., neural activity via EEG) in adults with DCD versus ADHD.

In fact, to date, explorations of neural activity via EEG versus behavioral performance have been rare for DCD versus control participants in general. To our knowledge, studies which have examined inhibition for individuals with DCD versus typically developing individuals have not yet included EEG to examine potential compensatory mechanisms, or in a more general brain-behavior comparison with a SST. However, there are some studies that examine inhibitory performance between individuals with ADHD versus typically developing individuals during a SST using EEG to capture event-related potentials (i.e., measurements of neural activity during discrete events; e.g., Bekker et al., 2005; MacLaren et al., 2007; Senderecka et al., 2012).

Among the studies examining inhibitory performance, those which included adult populations often found SST performance did not differ at the behavioral level for those with ADHD when compared to typically developing individuals, but variations have been found at the neural level (e.g., Bekker et al., 2005; MacLaren et al., 2007). More specifically, one study showed no differences on go trials, but revealed significantly longer SSRTs in the ADHD group (versus a typically developed individuals) coupled with significantly lower P300 ERPs (interpreted as an index of inhibition; Bekker et al., 2005). However, in another study, adults with ADHD did not differ in general behavioral performance on a simple SST compared to typically developing individuals, but instead showed significant differences in ERPs for P300 and N200 during stop trials (MacLaren et al., 2007). While the precise neural substrates of P300 and N200 have often been debated, both are thought to relate to aspects of inhibition, attention, and other executive functions in the context of a SST (Bekker et al., 2005; MacLaren et al., 2007; Huster et al., 2020). Taken together, these findings highlight the importance of testing the underlying neural responses to the SST in adult clinical populations. This may help elucidate the distinctions between those with ADHD and typically developing individuals.

### The Current Study

The present study examined both behavioral and neural levels of performance of participants with DCD, ADHD, DCD + ADHD, and typically developing control participants in a motor inhibition task. We aimed to improve the understanding of brain-behavior differences in these adult groups in order to better inform the co-occurrence of DCD and ADHD, as well as to highlight differences between the occurrence of DCD versus ADHD alone. We expected that, due to compensatory mechanisms, no differences would be present at the behavioral level in general go accuracy, and mean reaction times for all trial types (particularly: Go RT, RT of unsuccessful stop trials, and SSRT). We further hypothesized that behavioral compensation among adults would relate to more robust differences in the EEG signals between groups, and more specifically, that they would be present in components P300 and N200 in line with symptoms of ADHD, DCD, or both conditions versus typically developing adults.

Due to insufficient data in the DCD literature to make specific assumptions of the direction of amplitudes in P300 and N200 we aimed to examine effects reported in the literature comparing ADHD and typically developing individuals. Furthermore, we explore and report all differences found in the complete set of electrodes to build insights into patterns when comparing DCD and DCD + ADHD groups. In addition, we explored all possible distinctions between the ERPs in the DCD and ADHD groups. These comparisons provide important pilot evidence in relation to group differences in inhibition and related executive functioning processes as well.

## Materials and Methods

### Sample

A total of *N* = 59 participants were recruited at two sites (Germany and United Kingdom). Following EEG pre-processing and our criteria for the removal of outliers (see “EEG Pre-processing” and “Statistical Analysis” sections below), a final sample of *N* = 50 was included in the present analyses with the same participants across behavioral and neurophysiological levels (see Table 1). This sample included *n* = 30 from Germany and *n* = 20 from the United Kingdom. Overall participants were 67% female, 76% right handed, and *M* = 25.5 (*SD* = 7.9) years old. Groups included those with an existing diagnosis of ADHD (*n* = 9), DCD (*n* = 12), both ADHD and DCD (*n* = 7), and a control group (*n* = 22). In order to run more reliable analyses, we combined the participants from both sites to result in adequate group sizes (see Table 1).

**TABLE 1:** Group classification and testing location comparisons.

**Groups: overall (*N* = 50)**	**Sample size**	**Average ADC score**	**Average ASRS v.1 score**	
DCD	12	113.1 (14.1)	42.5 (9.3)	
ADHD	9	87.8 (12.0)	59.0 (9.0)	
DCD + ADHD	7	108.1 (11.7)	52.9 (11.7)	
Control	22	66.7 (12.8)	44.0 (8.2)	

**Participants from Germany (*n* = 30)**	**Sample size**	**Average ADC score**	**Average ASRS v.1 score**	

DCD	1	119.0	48.0	
ADHD	6	88.2	60.8	
DCD + ADHD	2	104.5	64.0	
Control	21	67.6	45.1	

**Participants from United Kingdom (*n* = 20)**	**Sample size**	**Average ADC score**	**Average ASRS v.1 score**	**Median MABC-2 percentile**

DCD	11	112.9	42.0	5th
ADHD	3	85.3	55.3	25th
DCD + ADHD	5	109.6	48.4	2nd
Control	1	50.0	22.0	63rd

**Overall group scores on the ADC and ASRS v.1 were compared via a one-way ANOVA. There was a significant effect of group on ADC score [F(3,46) = 38.37, p < 0.001]. Post hoc tests revealed all group comparisons were significant (p < 0.05) aside from the DCD and DCD + ADHD group comparison. There was also a significant effect by group on ASRS v.1 scores [F(3,45) = 7.78, p < 0.001]. Post hoc tests revealed this effect was driven by all group comparisons except for ADHD and DCD + ADHD; DCD and DCD + ADHD; DCD and the control group; DCD + ADHD and the control group (p > 0.05).**

In the clinical groups, all participants reported previous diagnoses of ADHD and/or DCD, and reported no history of brain damage or other developmental impairments (e.g., cerebral palsy). The control group reported no history of any psychiatric or other health conditions. Additionally, participants with ADHD were asked to not take ADHD-relevant medication on the day of the study session if they had the option. None of the participants included in the final sample reported taking such medication on the day of testing. The protocol was reviewed and approved by ethics committees at both sites (University of Mannheim and Oxford Brookes University).

### Measures

We administered the Adult Dyspraxia/DCD Checklist (ADC; Kirby et al., 2010), it yielded good reliability in the present study via Cronbach’s Alpha in overall scores (α = 0.950) and in its standard three subsections (A: difficulties in childhood, α = 0.918; B: current difficulties, α = 0.883; C, current difficulties manifested by others, α = 0.851). In addition, we compiled the Adult Self Report Screening (ASRS v.1) for ADHD (Kessler et al., 2005), which also yielded good reliability overall (α = 0.875) as well as in subsections A (adult ADHD symptom overview, α = 0.687) and B (adult ADHD specific symptom probes, α = 0.835).

### Stop-Signal Task Features

**FIGURE 1:**
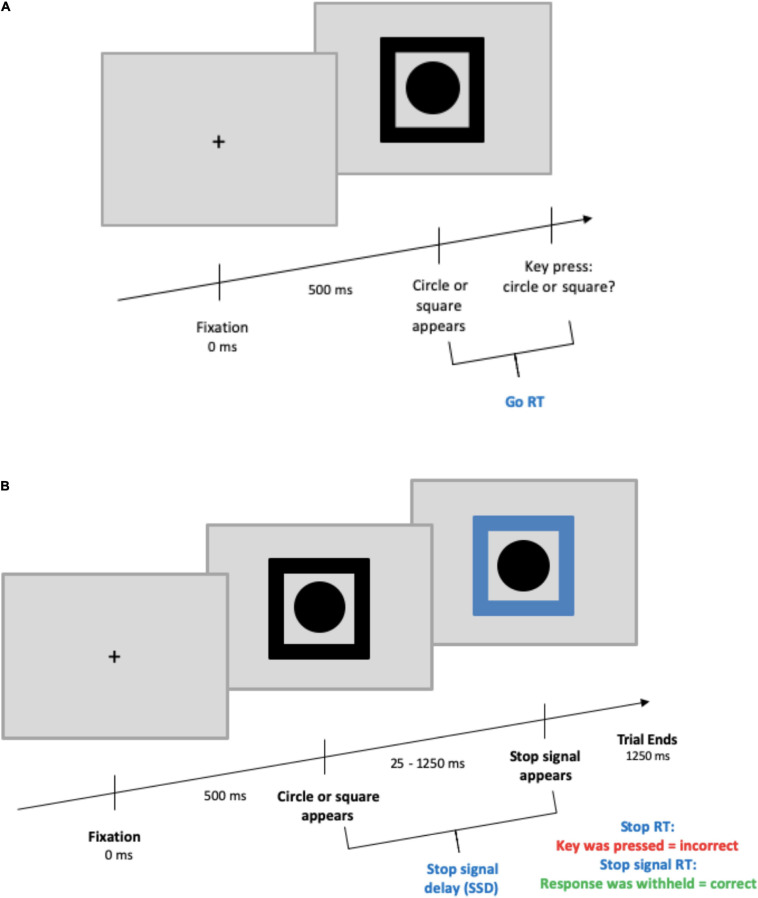
Stop-signal task design. **(A)** Depicts a go trial, **(B)** depicts a stop trial. For both trial types, the maximum time from the appearance of the circle/square to the end of a trial was 1,250 ms. The ITI was randomized between 375 and 625 ms and presented with a blank screen.

The SST began with a black fixation cross at the center of the screen appearing for 500 ms. Next, a black shape cue (circle or square) was presented in the center of the screen surrounded by a black frame. On go trials, participants needed to press the key corresponding to the shape shown (go stimulus; counterbalanced “c” or “v” keys). On stop trials (25% of all trials) participants were instructed to refrain from pressing a key. Here, the black frame was replaced by a blue frame after a variable delay known as stop-signal delay (SSD). The SSD ranged from 250 to 1,250 ms with an adaptive up-down staircase method (Levitt, 1971) based on performance in steps of ±25 ms. The SSD was increased with successful inhibition and decreased with failed inhibition to maintain a 50% stop accuracy rate for each participant. Participants were informed of this tracking procedure and subsequently were to respond as fast and accurately as possible without waiting for the stop-signal to appear. The entire SST was presented against a gray background, and the SST was based on that of Gajsar et al. (2020; see Figure 1).

Participants completed a total of 768 trials across six blocks with open-ended breaks after each block. There were 128 trials per block, with 64 trials (48 go, 16 stop) per shape. The stimuli were presented on computers with MATLAB^®^ (The MathWorks, Inc., Natick, MA, United States) using the Psychtoolbox extensions (Kleiner et al., 2007) on a 16 inch screen in Germany and a 24 inch screen in the United Kingdom. Viewing distances were approximately 5 and 3 feet, and visual angles of 43 and 67°, respectively.

Following recent guidelines on the SST set out by Verbruggen et al. (2019), this visual stop-signal task was designed with all recommended features aside from practice trials and block-based feedback of performance. In lieu of practice trials, the researchers checked in with participants after the first block to ensure that, to their knowledge, they completed the task properly. Block-based feedback was not included in order to reduce any external influences on performance given the novel DCD and DCD + ADHD groups.

### Procedure

Prior to the main task, participants completed the ASRS v.1 for ADHD (Kessler et al., 2005), the ADC (Kirby et al., 2010), as well as questions about their demographics and health history. Participants in the United Kingdom also completed the MABC-2 age band 3 (Henderson et al., 2007). These measures were used to confirm preexisting diagnoses of DCD and/or ADHD for group assignment, and to ensure members of the DCD group did not have signs of ADHD, and vice versa (see Table 1). Next, participants were prepared for EEG measurement and then completed the stop-signal task. As a part of other studies, a subgroup of participants completed the SST and additional computer tasks in random order.

### EEG Data Pre-processing

A 64 channel system was utilized for electrophysiological recoding in a standard 10–20 system. FCz was used as the reference electrode and AFz as the ground (for setup see Gerdes et al., 2013). Scalp impedances were maintained at 15 kΩ and below. Recordings were made at a sampling rate of 1 kHz at one testing location and 500 Hz at another; therefore, all data were adjusted to a 500 Hz sampling rate. The average of all channels was used as the reference in the data processing phase. A 50 Hz notch filter was implemented to remove any confounding high frequency noise. Moreover, a band pass filter was set from 0.5 to 70 to reflect the more cautious approach used in other studies with a SST and related clinical groups (e.g., Senderecka et al., 2012). Eye blink artifacts were removed with an Independent Component Analysis (ICA). Segments during responses were set with windows from −100 ms before the event to 400 ms after the event. For some participants, electrodes with uninterpretable signals were corrected with a topographic interpolation. An average of *M* = 0.94 (*SD* = 1.21, Range: 0–5) electrodes per participant needed to be corrected with topographical interpolations.

Trigger recoding was performed for all participants to tag trials as correct versus incorrect. In this process, three participants had insufficient or poor quality EEG data for which triggers could not be recoded, such that the recalculated triggers displayed a significant discrepancy between the original and new fixation trigger time points. This led to the exclusion of *n* = 3 participants.

Following other studies that have used the SST with ADHD and control groups (e.g., Kok et al., 2004; Ramautar et al., 2006; Johnstone et al., 2007; Senderecka et al., 2012), electrodes were explored with time windows set at 200 ms −310 ms for N200, and 230 ms −400 ms for P300. Epochs were defined at 100 ms pre-stimulus and 400 ms post-stimulus. We report any significant differences in amplitudes for individual electrodes.

### Statistical Analysis

Participants were excluded if their average reaction times on go trials were larger than unsuccessful stop trial reaction times, thereby violating the independence assumption of the race model of the SST (Verbruggen et al., 2019). Following Verbruggen et al. (2019), mean RTs in this comparison included all trials with a key press (i.e., responses may also be incorrect or premature. This led to the removal of *one* participant with co-occurring DCD and ADHD from consecutive analysis. In an additional step, within-subjects outliers, i.e., extreme trial-level raw go RT and unsuccessful stop RT scores, were removed (on average, 4% an 33%, respectively) to exclude premature and late responses based on the criteria recommended by Leys et al. (2013; i.e., median ± 2.5 × absolute median score).

Average go reaction times were then computed for correct go trials and unsuccessful stop reaction times, with the latter including only responses in which a key was incorrectly pressed. The stop-signal reaction time (SSRT), or the estimated amount of time required to inhibit a response about to be executed, represents response latencies that were estimated for stop trials (i.e., in which a key was not pressed). The SSRTs were calculated with the block-based integration method (Verbruggen et al., 2013; see Gajsar et al., 2020 for a detailed description of this procedure). However, mean SSDs involved in estimating SSRTs were used to align with actual screen presentation times and are referred to simply as “SSDs”; see Verbruggen et al., 2019). This method is preferable when including clinical groups, such as individuals with ADHD (Verbruggen et al., 2013

Following the calculations above, performance was further screened to increase reliability by removing outlying individuals with sub-optimal performance based on the lenient outlier criteria set by Congdon et al. (2012), slightly adapted for clinical populations (see below). Based on this, participants were excluded if, on average, they violated one or more of the following criteria: (1) a proportion of successful inhibition on stop trials greater than 25% and less than 75%, (2) a proportion of go response greater than 60%, (3) an estimated SSRT which is positive and greater than 50 ms, and (4) a proportion of go errors less than 15%. The fourth criterion required slight modification to 15% (instead of 10%) to account for outliers in the DCD + ADHD group. Screening for these four criteria resulted in the removal of an additional *n* = 5 participants (*n* = 3 in control group; *n* = 1 with DCD; *n* = 1 with ADHD).

Behavioral and neurophysiological results were compared with between-subjects One-Way ANOVAs and Tukey’s LSD *post hoc* tests. We also conducted independent samples *t*-tests to compare the amplitudes of ERPs between participants with DCD and ADHD in particular. Kolmogorov–Smirnov tests revealed the control group had a non-normal distribution (*D* = 0.20, *p* = 0.02) for SSRTs, which prevents its comparison to the other groups for correct stop trials. All aforementioned group comparisons held before and after outlier removal. Statistical analysis was conducted in R (v. 3.6.2).

## Results

### Group Confirmation

As mentioned, participants were grouped based on their reported diagnostic history for DCD, ADHD, both conditions (DCD + ADHD), or no health conditions, and this was confirmed by self-reported symptoms. The average ADC scores for each group from highest to lowest were: DCD (*M* = 113.1, *SD* = 14.1), DCD + ADHD (*M* = 108.1, *SD* = 11.7), ADHD (*M* = 87.8, *SD* = 12.0), and the control group (*M* = 66.7, *SD* = 12.8). Scores of 90 and above signify probable DCD, and scores over 80 signal potential risk for DCD (Kirby et al., 2010). The ASRS v.1 scores from highest to lowest per group were: ADHD (*M* = 59.0, *SD* = 9.0), DCD + ADHD (*M* = 52.9, *SD* = 11.7), controls (*M* = 44.0, *SD* = 8.2), and the DCD group (*M* = 42.5, *SD* = 9.3) where a score of 47 or higher is indicative of likely ADHD. Classifications by group and testing site are listed in Table 1.

### Stop-Signal Task Behavioral Parameters

Stop accuracy was not significantly different between groups, however, there was a significant difference for groups on go accuracy [*F*(3,44) = 4.15, *p* = 0.011]. As revealed in *post hoc* testing, this difference was driven by a significant lower accuracy in the DCD + ADHD (*M* = 0.93, *SD* = 0.19) group compared to the control group (*M* = 0.97, *SD* = 0.11). The average reaction times for go trials, unsuccessful stop trials, and correct (i.e., successful) stop trials (SSRTs) were not significantly different between groups, whereby SSRTs were only compared between clinical groups (see Table 2 for the descriptive statistics of the dependent measures of SST performance for all participants).

**TABLE 2:** Descriptive statistics of dependent measures for stop-signal task behavioral data overall and per group.

**Dependent Measure**	**Group**	***M***	***SD***	**Range**
Probability of go omissions (no response)	DCD	0.02	0.03	0–0.07
	ADHD	0.04	0.04	0–0.14
	DCD + ADHD	0.03	0.04	0–0.1
	Control	0.02	0.02	0–0.08
	*Overall*	0.03	0.03	0–0.14
Probability of choice errors on go trials	DCD	0.04	0.03	0–0.1
	ADHD	0.04	0.04	0–0.13
	DCD + ADHD	0.07	0.05	0.02–0.14
	Control	0.03	0.02	0–0.09
	*Overall*	0.04	0.03	0–0.14
RT on go trials (mean)	DCD	663.64	107.25	491.25–842.05
	ADHD	618.46	167.22	402.91–837.31
	DCD + ADHD	654.59	197.17	496.53–988.59
	Control	672.49	151.75	407.1–904.31
	*Overall*	656.73	147.31	402.91–988.59
Intra-subject variability of correct go trials	DCD	133.40	35.80	67.43–191.7
	ADHD	120.17	47.27	49.42–192.59
	DCD + ADHD	114.68	35.26	80.69–167.44
	Control	122.37	39.98	54.47–184.19
	*Overall*	122.76	39.12	49.42–192.59
Probability of responding on a stop trial	DCD	0.50	0.03	0.45–0.59
	ADHD	0.49	0.02	0.45–0.51
	DCD + ADHD	0.48	0.03	0.43–0.51
	Control	0.48	0.03	0.44–0.56
	*Overall*	0.49	0.03	0.43–0.59
Average stop-signal delay	DCD	377.79	124.27	143.18–574.17
	ADHD	359.58	155.08	170.18–557.71
	DCD + ADHD	374.36	204.97	141.18–674.2
	Control	413.00	148.26	141.17–656.19
	*Overall*	389.00	147.96	141.17–674.20
Stop-signal reaction time	DCD	286.24	72.18	228.29–479.07
	ADHD	256.02	30.75	226.54–320.42
	DCD + ADHD	275.51	47.19	231.08–358.23
	Control	254.34*	43.42	200.4–413.9
	*Overall*	264.13	50.29	200.40–479.07
RT of go responses on unsuccessful stop trials	DCD	569.30	70.75	458.94–690.85
	ADHD	545.09	149.42	373.3–811.73
	DCD + ADHD	575.60	188.01	424.23–903.2
	Control	583.06	129.95	379.44–813.99
	*Overall*	570.77	126.75	373.30–903.20

Measures listed as recommended by Verbruggen et al. (2019), including measures for each group and overall. The SSRT was not estimated for n = 5 participants exhibiting suboptimal performance. An additional table is included in Supplementary Materials listing descriptive measures for these participants. When outliers were removed on a trial-by-trial basis, group level differences did not change. *Not normally distributed.

### ERP Results

#### N200: All Group Comparisons

**FIGURE 2:**
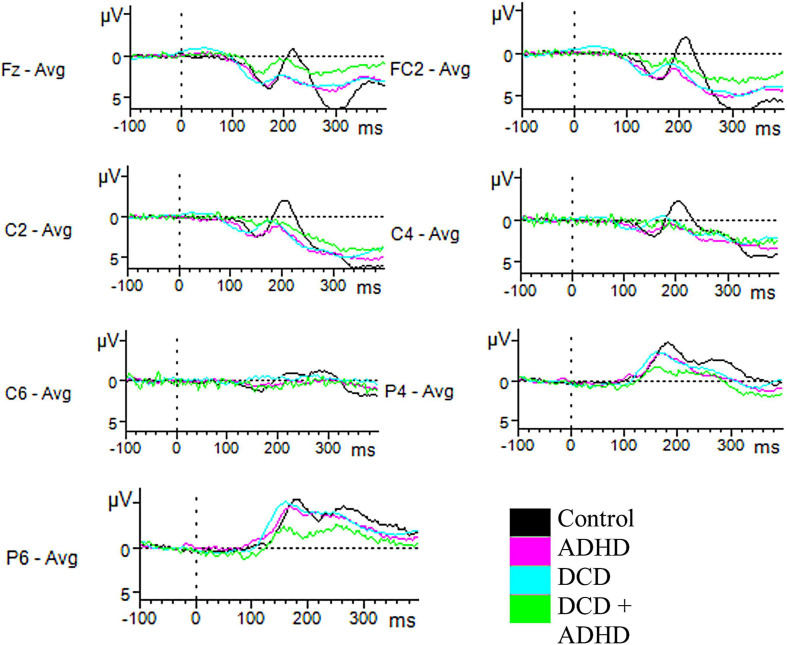
Successful-stop ERPs. Depicted are all electrodes with significant differences in amplitudes for N200 and P300 for correct stop trials. Time windows were set at 200–310 ms for N200, and 230–400 ms for P300. For N200, significant differences in amplitudes in C2, C4, and FC2 were driven by differences in the ADHD and control groups, while differences for C6 were driven by differences between the control and DCD groups, and the control and ADHD groups. Differences in P4 and P6 were driven by differences in the control and DCD + ADHD groups. For P300, a significant difference between groups was indicated for electrode Fz, driven by the difference between the DCD + ADHD and control groups.

**FIGURE 3:**
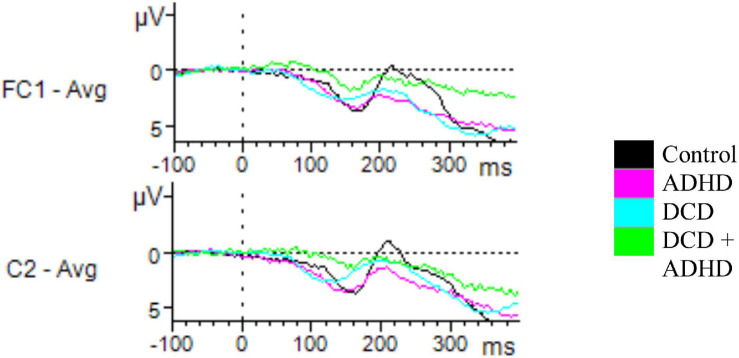
Unsuccessful stop ERPs. Depicted are all electrodes with significant differences in amplitudes for N200 and P300 for incorrect stop trials. Time windows were set at 200–310 ms for N200, and 230–400 ms for P300. For N200, a significant effect of group on amplitude was found for C2, driven by a difference between the ADHD and control groups. For P300, a significant difference was found for FC1, driven by the DCD + ADHD and control groups.

For component N200, there were amplitudes of several electrodes for which a significant group effect was found. This included C2 [*F*(3,46) = 3.47, *p* = 0.024], C4 [*F*(3,46) = 3.78, *p* = 0.017], C6 [*F*(3,46) = 3.15, *p* = 0.034], FC2 [*F*(3,46) = 3.25, *p* = 0.030], P4 [*F*(3,46) = 3.00, *p* = 0.040], and P6 [*F*(3,46) = 3.93, *p* = 0.014] during successful inhibition (correct stop trials). *Post hoc* tests revealed several of these differences were driven by the distinction in amplitudes between the ADHD and control groups (C2, ADHD: *M* = 0.71, *SD* = 1.74, Control: *M* = −2.27, *SD* = 2.88, *p* = 0.016; C4, ADHD: *M* = −0.28, *SD* = 1.75, Control: *M* = −3.04, *SD* = 2.69, *p* = 0.018; FC2, ADHD: *M* = 1.10, *SD* = 1.59, Control: *M* = −2.12, *SD* = 3.32, *p* = 0.020). A further difference in C6 was driven by marginally significant differences between the DCD and ADHD groups (DCD: *M* = −2.93, *SD* = 2.31, ADHD: *M* = −1.02, *SD* = 0.94, *p* = 0.056) and ADHD and control groups (Control: *M* = −2.64, *SD* = 1.43, *p* = 0.076; see Figure 2). Furthermore, an effect in amplitudes for electrodes P4 and P6 was driven by differences between the DCD + ADHD and control groups (P4, ADHD: *M* = −2.82, *SD* = 1.44, Control: *M* = −5.29, *SD* = 2.03, *p* = 0.048; P6, ADHD: *M* = −3.86, *SD* = 1.82, Control: *M* = −6.78, *SD* = 2.08, *p* = 0.010). Finally, one electrode was implicated in unsuccessful inhibition (incorrect stop trials): C2 [*F*(3,46) = 3.01, *p* = 0.040], for which *post hoc* testing revealed differences between the ADHD (*M* = 0.59, *SD* = 1.14) and control groups (*M* = −2.44, *SD* = 3.43, *p* = 0.037; see Figure 3).

#### P300: All Group Comparisons

When considering component P300, there was a significant effect on the amplitudes of various electrodes based on group, including Fz during successful inhibition [*F*(3,46) = 3.06, *p* = 0.038], FC1 during unsuccessful inhibition [*F*(3,46) = 3.05, *p* = 0.038], and T7 during correct go trials [*F*(3,46) = 3.39, *p* = 0.026]. *Post hoc* testing via Tukey’s HSD revealed these findings were driven by differences between the DCD + ADHD and control groups (Fz, DCD + ADHD: *M* = 4.83, *SD* = 2.98, Control: *M* = 8.89, *SD* = 4.57, *p* = 0.092; FC1, DCD + ADHD: *M* = 4.95, *SD* = 3.01, Control: *M* = 9.37, *SD* = 4.58, *p* = 0.047; T7, DCD + ADHD: *M* = 2.63, *SD* = 2.48, Control: *M* = 0.32, *SD* = 1.68, *p* = 0.016). However, in Fz this group difference was just marginally significant (see Figures 2, 3).

#### Exploratory Comparisons: DCD Versus ADHD

No significant differences between the ADHD and DCD groups were found during go trials, however, for P300 and N200 on stop trials, there were several noteworthy differences between the DCD and ADHD groups in particular. During successful inhibition, significantly higher amplitudes were present in P300 for the DCD group compared to the ADHD group in fronto-temporal electrodes AF7 [*t*(19) = 3.29, *p* = 0.004] (DCD: *M* = 4.30, *SD* = 1.84; ADHD: *M* = 1.88, *SD* = 1.40), F7 [*t*(19) = 2.99, *p* = 0.008] (DCD: *M* = 3.91, *SD* = 1.87; ADHD: *M* = 1.66, *SD* = 1.46), and FT7 [*t*(19) = 2.96, *p* = 0.008] (DCD: *M* = 3.32, *SD* = 1.19; ADHD: *M* = 1.59, *SD* = 1.49). During unsuccessful inhibition, the DCD group had heightened peaks in activation for P300 compared to the ADHD group for electrodes AF7 [*t*(19) = 2.44, *p* = 0.025] (DCD: *M* = 4.54, *SD* = 2.60; ADHD: *M* = 2.08, *SD* = 1.76), F7 [*t*(19) = 3.05, *p* = 0.008] (DCD: *M* = 5.27, *SD* = 1.82; ADHD: *M* = 2.64, *SD* = 2.13), and T7 [*t*(19) = 2.56, *p* = 0.018] (DCD: *M* = 3.00, *SD* = 1.20; ADHD: *M* = 1.46, *SD* = 1.54).

In addition, several differences in central and fronto-central electrodes were present between the DCD and ADHD groups for N200, including FC2 [*t*(19) = 2.29, *p* = 0.034] (DCD: *M* = −0.96, *SD* = 2.31; ADHD: *M* = 1.10, *SD* = 1.59), FC6 [*t*(19) = 2.60, *p* = 0.022] (DCD: *M* = −2.01, *SD* = 2.14; ADHD: *M* = −0.33, *SD* = 0.57), C2 [*t*(19) = 2.13, *p* = 0.047] (DCD: *M* = −1.14, *SD* = 2.13; ADHD: *M* = 0.71, *SD* = 1.74), and C6 [*t*(19) = 2.32, *p* = 0.032] (DCD: *M* = -2.93, *SD* = 2.31, ADHD: *M* = −1.02, *SD* = 0.94) during successful inhibition. Furthermore, there were significant differences between groups for N200 peaks in several electrodes during unsuccessful inhibition, including C1 [*t*(19) = 2.42, *p* = 0.027] (DCD: *M* = −0.93, *SD* = 2.61; ADHD: *M* = 1.17, *SD* = 1.30), and C2 [*t*(19) = 2.31, *p* = 0.034] (DCD: *M* = −1.29, *SD* = 2.48; ADHD: *M* = 0.59, *SD* = 1.14). In all aforementioned differences in N200, the DCD group showed a significantly more negative amplitude than the ADHD group.

## Discussion

This study lays important groundwork in the DCD literature by examining endophenotypic overlaps and differences in executive functions. This novel design includes a stop-signal task and neurophysiological measurements among adults with DCD, ADHD, both conditions, and typically developing individuals (control group). As expected, the behavioral results showed few differences between all groups. One difference was present in that the go accuracy of individuals with co-occurring DCD + ADHD was lower than for typically developing individuals. Also in line with our expectations, several differences were found as indexed by ERPs between all groups, with additional differences found in direct comparison of the DCD versus ADHD groups. The latter comparison showed that many electrodes had significantly different amplitudes for components P300 and N200 between the DCD and ADHD groups. There were several electrodes, especially in central locations, which indicated differences in amplitude for P300 and N200 components on stop trials. These differences were not present for go trials which signifies that the general presence of the stop-signal may activate a specific neurophysiological response. This is in line with other research comparing those with ADHD to typically developing individuals in various age groups (e.g., Kok et al., 2004; Ramautar et al., 2006; Johnstone et al., 2007; Senderecka et al., 2012). While N200 is often viewed as an index of cognitive flexibility in typically developing individuals, P300 is viewed as an index of attention, and both have implications for attention and response inhibition in the SST (Chikara et al., 2018; Huster et al., 2020). Given that there were several key differences in these components in the present study, mechanisms of attention, response inhibition, and/or cognitive flexibility seem to differ for DCD and ADHD.

Aside from go accuracy, those with combined DCD + ADHD did not perform significantly different from all other groups at the behavioral level. This result aligns with previous studies of adults with DCD or ADHD compared to typically developing individuals where no differences in behavioral results were present between groups (Bekker et al., 2005; MacLaren et al., 2007; He et al., 2018a). Due to high demands on inhibitory performance in the SST (see Rubia et al., 2001), these results are unlikely to be accounted for by a floor effect in the clinical group, as they perform similarly to the control groups. Should our result hold in larger samples, it would indicate adults with DCD and/or ADHD can compensate in order to perform as well as typically developed adults in overt responses on the stop-signal task (i.e., inhibitory control and related engagement of attention).

The general absence of differences in overt behavior emphasizes the lack of diagnostic power of typical measures of accuracy and reaction times regarding differential diagnosis of DCD or ADHD in adults. Differences in task performance on other inhibition tasks (e.g., Stroop task) have previously been considered as indicators of possible neurological differences among those with ADHD in particular, but similarly are not sufficient for a diagnosis (Homack and Riccio, 2004). This could be due to effortful compensation in adults, or more broadly because inhibitory control is extremely complex and can be gaged differently by various inhibition tasks (Mirabella, 2021).

Evidence at the neural level indicates there may be unique neural signatures in evoked potentials between the DCD and ADHD groups, supporting findings of other studies (Langevin et al., 2014; McLeod et al., 2014). In the current study, several differences in activation were present in the N200 component for DCD versus ADHD groups on stop trials, especially in central regions during successful inhibition (i.e., correct performance on a stop trial). Interestingly, the amplitude for the DCD group was consistently larger than in the ADHD group regardless of successful versus unsuccessful inhibition. This provides evidence of separate ways of engaging attentional and inhibitory resources between these groups to achieve the same overt response.

Importantly, while behavioral performance did not differ between groups in the majority of parameters, inhibition and task engagement are not necessarily employed with the same underlying neural mechanisms across groups. It is unclear if these underlying mechanisms also translate to increased effort and/or fatigue, but this should be explored in more detail in future research. Given that compensation is more readily achieved when a task is less complex for DCD groups in particular (e.g., Pratt et al., 2014), it may also be the case that the task was too complex for the DCD + ADHD group with a higher symptom load.

Overall, the present study provides several novel contributions to the DCD and ADHD literature. First, to our knowledge it is the first study to compare inhibition performance between adult participants with DCD versus ADHD using a SST, as well as group comparisons between those with DCD, ADHD, DCD + ADHD, and typically developing adults. It is also the first study to incorporate an additional layer of EEG measurement to examine such group differences during the inhibition of a motor response to a visual cue. While most research on symptom differentiation relies on self-report questionnaires (Eisenbarth et al., 2008), some studies have also investigated endophenotypes in DCD or ADHD via motor performance or attentional performance exclusively with single-occurring DCD or ADHD compared to typically developing individuals, but rarely both. Third, investigations on adults with both ADHD and DCD are extremely rare. Therefore, examining this population can provide researchers with important insights into the endophenotypes and clinical picture of ADHD and DCD in adults.

### Limitations and Future Directions

There are several limitations of the present study which should be considered. First, the sample sizes were small, in particular those of the clinical sub-groups. A normality check performed due to unequal group sizes indicated SSRTs of the typically developing (control) group were not normally distributed, even though the typically developing adults comprised of the largest group of the four in this study. Nonetheless, normality checks for other groups passed. Small and unequal group sizes are not a new problem in DCD and ADHD research, especially when involving neuroscientific measurements (e.g., Querne et al., 2008; McLeod et al., 2014). In that respect, our sample sizes are in the range of previous studies. Future research should replicate this initial study with larger groups. While some shortcomings are expected in pilot testing, the present study is nonetheless important and novel by including diverse groups of adults with DCD, ADHD, DCD + ADHD, and typically developing individuals.

A second limitation is the duration of the study sessions. Participants completed 768 trials of the stop-signal task, and this took most participants around 40 min. While the participants all took breaks between blocks, this can still be straining, especially to participants with difficulties in sustaining attention. There is evidence of this for the DCD + ADHD group in particular, who performed significantly worse than typically developing individuals in go accuracy, which are the least complex trials in the task. This could be explained by a difficulty managing competing cognitive resources of the task (e.g., executive versus inhibitory performance) and could be related to a higher symptom load in the DCD + ADHD group.

Another limitation is that the mean performance measures (i.e., accuracy and reaction-time) did not reveal many substantial differences but they do not confirm a null hypothesis for behavioral results in the present study. Our main focus was to examine if neurophysiological differences would differ between groups for components linked to attention and motor performance with the expectation that behavioral data would reveal no differences, as observed in other studies with related samples (e.g., Bekker et al., 2005; MacLaren et al., 2007; He et al., 2018a). More robust cognitive models could potentially reveal more subtle differences between groups not detectable in mean RTs or accuracy. Future research should consider the use of cognitive models (e.g., the diffusion model, Ratcliff and McKoon, 2008; White et al., 2010) in behavioral data for those with DCD, ADHD, and both conditions. In addition, these models might better differentiate symptoms present in both DCD and ADHD related to visuo-motor integration deficits in particular (Kurdziel et al., 2015; Nobusako et al., 2018). Future research should also consider the utilization of a task with visuo-motor components in order to better understand the potential differences between DCD and ADHD in visuo-motor integration and inhibition, and how it is cognitively employed in relation to profiles of each disorder.

Additional limitations include the participant demographics (e.g., majority female, differing equipment at testing locations). Also, participants were recruited in two different sites. While the laboratory equipment and practices of the researchers remained as consistent as possible, some differences between the testing locations existed, such as the visual angles for the task. On the other hand, it may be an advantage that multinational groups of individuals may help to generalize the findings and is a method that has been used before with DCD (e.g., United Kingdom and Israel, Kirby et al., 2010). We argue that the benefit of increasing the sample size outweighs the possibly subtle differences in laboratories. Nonetheless, future research should replicate these findings with a larger population and balanced groups, but also across additional test sites and cultures.

Another limitation is the lack of specificity in N200 and P300 in general. ERP components are broad constructs, but are useful in the present study to indicate several processes in executive functioning (e.g., attention, action, inhibition; Huster et al., 2020). A source analysis for P300 and N200 components (e.g., Moores et al., 2003; Huster et al., 2010; Hong et al., 2017) should be considered in dedicated future research with larger samples to understand the localization of processes involved in executive functioning during a SST. Given that the present study aimed to identify any potential differences in ERPs as foundational evidence for future research, we reported all relevant electrode sites. However, it also is essential to replicate amplitude differences with *a priori* hypotheses in larger samples with respect to specific electrodes or sensor sites to account for potential false positives by testing many sensors.

Finally, future research should consider approaches with multiple levels of neurophysiological assessment, including neuroimaging and neurostimulation. Recent pilot work using TMS in adults with DCD versus typical adults has shown to be promising in identifying correlates of motor symptoms of DCD at the neural level (He et al., 2018b; Hyde et al., 2019). Combined approaches between some of these methods, such as EEG and TMS, could be particularly effective in disentangling DCD and ADHD symptoms at the neural level.

The present study provides an important initial step in identifying underlying neural processes which may not be reflected in behavioral performance of adults with DCD and ADHD. In addition to further work needed to confirm our findings in behavioral and electrophysiological differences in adults with DCD and/or ADHD during the SST, there is still a need for more research on other relevant endophenotypes in DCD and ADHD which can be compared in other paradigms (e.g., attention; Conzelmann et al., 2010). Also, we have not identified the specific compensatory mechanisms that were used (e.g., adaptive versus maladaptive), or if they were based on motor or executive functioning processes. So far we can only assert that there is evidence in the present study that compensation is being employed by participants in clinical groups. Future research should consider identifying more specific features of compensation mechanisms for those with DCD or ADHD, for example, in different age groups with longitudinal designs.

## Conclusion

This pilot study innovatively demonstrated that inhibitory control may be a relevant endophenotype at behavioral and neurophysiological levels for the differentiation of DCD, ADHD, and co-occurring DCD and ADHD. Crucially, we identified patterns of varying P300 and N200 amplitudes, suggesting there are unique executive mechanisms utilized to inhibit a motor response between groups. At the same time, our results (i.e., group differences in ERPs but largely similar behavioral performance between groups), may reflect the potential strength and success of compensatory mechanisms in individuals with DCD and/or ADHD. This study serves as an important foundation for future explorations into the overlapping executive functioning processes in DCD and ADHD.

## Data Availability Statement

The raw data supporting the conclusions of this article will be made available by the authors, without undue reservation, to any qualified researcher.

## Ethics Statement

The studies involving human participants were reviewed and approved by University of Mannheim and Oxford Brookes University Ethics Committees. The patients/participants provided their written informed consent to participate in this study.

## Author Contributions

EM, MM, KW, MZ, and GA were involved in the conception or design of the work, data interpretation, critical revision of article, and approval of the version submitted for publication. EM tested all participants, conducted data analysis of measures, behavioral data, electrophysiological data, conducted the literature review, and formulated the initial draft of the manuscript. MM developed the stop-signal task used in this study in Matlab and wrote R scripts for behavioral analysis and trigger recoding. MZ provided funding for participants recruited in Germany. KW provided funding for participants recruited the United Kingdom. All authors contributed to the article and approved the submitted version.

## Conflict of Interest

The authors declare that the research was conducted in the absence of any commercial or financial relationships that could be construed as a potential conflict of interest.

## Supplementary Material

FIGURE S1 Descriptive measures of participants removed from behavioral analysis (*n* = 5).
