# Supplementary material for: Evoked Potentials Differentiate Developmental Coordination Disorder From Attention-Deficit/Hyperactivity Disorder in a Stop-Signal Task: A Pilot Study
Source: Front Hum Neurosci. 2021 Mar 11;15:629479. doi: 10.3389/fnhum.2021.629479 (PMC7990764; doi:10.3389/fnhum.2021.629479)
Supplement: FIGURE S1 — Descriptive measures of participants removed from behavioral analysis (n = 5). [file Table_1.docx]

**Supplemental Materials**

Table 1. Descriptive measures of participants removed from behavioral analysis (*n* = 5)

| Dependent Measure | M | SD | Range |
| --- | --- | --- | --- |
| Probability of go omissions (no response) | 0.22 | 0.36 | 0.02 - 0.86 |
| Probability of choice errors on go trials | 0.61 | 0.38 | 0.15 - 0.99 |
| RT on go trials (mean) | 707.53 | 240.59 | 534.85 - 1121.21 |
| Intra-subject variability of correct go trials | 136.85 | 86.15 | 73.07 - 287.79 |
| Probability of responding on a stop trial | 0.41 | 0.18 | 0.08 - 0.5 |
| Average stop-signal delay | 525.15 | 354.17 | 263.71 - 1100.07 |
| Stop-signal reaction time | 180.28 | 194.89 | -165.63 - 295.77 |
| RT of go responses on unsuccessful stop trials | 620.35 | 164.25 | 495.22 - 815.61 |

*Note*: These data are reported following the recommendation of Verbruggen et al., (2019).
